# Effect of a controlled lifestyle intervention on medication use and costs: The Healthy Lifestyle Community Program (cohort 2)

**DOI:** 10.1177/02601060231164665

**Published:** 2023-03-20

**Authors:** Ragna-Marie Kranz, Carmen Kettler, Corinna Anand, Christian Koeder, Sarah Husain, Nora Schoch, Anette Buyken, Heike Englert

**Affiliations:** 1 Institute of Nutrition, Consumption and Health, Faculty of Natural Sciences, 26578 Paderborn University, Paderborn, Germany; 2 Department of Food Nutrition Facilities, University of Applied Sciences Muenster, Muenster, Germany

**Keywords:** Controlled intervention trial, non-communicable diseases, diabetes, hypertension, dyslipidemia, lifestyle intervention, medication, health economics

## Abstract

**Background:** Establishing a healthy lifestyle has a great potential to reduce the prevalence of non-communicable diseases (NCDs) and their risk factors. NCDs contribute immensely to the economic costs of the health care system arising from therapy, medication use, and productivity loss. **Aim:** The aim of this study was to evaluate the effect of the Healthy Lifestyle Community Program (cohort 2; HLCP-2) on medication use and consequently on medication costs for selected NCDs (diabetes, hypertension, and dyslipidemia). **Methods:** Data stem from a 24-month non-randomised, controlled intervention trial aiming to improve risk factors for NCDs. Participants completed questionnaires at six measurement time points assessing medication use, from which costs were calculated. The following medication groups were included in the analysis as NCD medication: glucose-lowering medications (GLM), antihypertensive drugs (AHD) and lipid-lowering drugs (LLD). Statistical tests for inter- and intra-group comparison and multiple regression analysis were performed. **Results:** In total, 118 participants (intervention group [IG]: *n* = 79; control group [CG]: *n* = 39) were considered. Compared to baseline medication use decreased slightly in the IG and increased in the CG. Costs for NCD medication were significantly lower in the IG than in the CG after 6 (*p* = 0.004), 12 (*p* = 0.040), 18 (*p* = 0.003) and 24 months (*p* = 0.008). After multiple regression analysis and adjusting for confounders, change of costs differed significantly between the groups in all final models. **Conclusion:** The HLCP-2 was able to moderately prevent an increase of medication use and thus reduce costs for medication to treat NCDs with the greatest impact on AHD.

**Trial registration:**

German Clinical Trials Register DRKS (www.drks.de; reference: DRKS00018775).

## Introduction

Approximately 70% of all deaths worldwide are related to non-communicable diseases (NCDs), the most common being cardiovascular diseases, cancer, chronic respiratory disease and diabetes. NCDs and their risk factors can be effectively prevented through lifestyle changes such as a healthy diet, sufficient physical activity, smoking cessation, stress management and regulation of body weight (WHO, 2020). A plant-based diet rich in dietary fibre, whole grains, and unsaturated fatty acids and low in animal products, salt and saturated fatty acids is the key characteristic of a healthy lifestyle (Jayedi et al., 2020). Interventions that follow a community-based approach are recommended for the prevention and management of NCDs as they address patientś needs and are shown to be effective in improving NCD risk profile (American Diabetes Association, 2019; Soltani et al., 2021; Sun et al., 2017). In their systematic review and meta-analysis, Soltani et al. (2021) examined 48 community-based intervention studies that addressed cardiovascular disease prevention using cardiometabolic risk factor reduction through lifestyle changes. They showed that community-based programs are effective in lowering systolic and diastolic blood pressure, LDL as well as total cholesterol, triglycerides and fasting glucose (Soltani et al., 2021). Better blood and vital parameters can lead to a reduction of prescribed medication, which has several positive effects such as cost savings, reduced side effects or a better quality of life (Burke et al., 2005).

In addition to the individual health consequences, a rising prevalence of NCDs due to an unhealthy lifestyle leads to an alarming increase in costs for the health care system (Bloom et al., 2011; Ding et al., 2016). For instance, the global expenses of diabetes could reach US$ 745 billion by 2030, while the worldwide costs of cardiovascular diseases could exceed US$ 1 trillion by 2025, which corresponds to an 22% increase since 2010. Here, direct costs including prescribed medication, treatment and care account for the largest share (Bloom et al., 2011). Lifestyle intervention programs with a community-based approach are recommended to reduce healthcare costs in order to curb the burden of NCDs (American Diabetes Association, 2019; Gaziano, 2007; Jacobs-van der Bruggen et al., 2007).

Studies in clinical settings have already demonstrated that lifestyle intervention programs can lead to a reduction in medication use for NCDs, especially in antidiabetic medication (Basterra-Gortari et al., 2019; Espeland et al., 2014; Hamdy and Carver, 2008; Johansen et al., 2017; The Look AHEAD Research Group, 2007). However, there are only a few studies analysing the medication use using a community-based approach without taking into account medication-associated costs (Connolly et al., 2017; Englert et al., 2012).

This research gap is addressed by the following health economic evaluation of the Healthy Lifestyle Community Program (cohort 2; HLCP-2). The objective was to test whether the lifestyle intervention would lead to a reduction in medication use and thus to a decrease in medication-associated health care costs compared to baseline and control. The HLCP-2 is a non-randomised, controlled lifestyle intervention using a community-based approach and was implemented in a rural population in Germany. The analysis focuses on drugs used to treat NCDs, including antihypertensive drugs (AHD), glucose-lowering medications (GLM), and lipid-lowering drugs (LLD). To the best of our knowledge, no previous article has evaluated the effect of a community-based lifestyle intervention on medication use and costs for NCD medication.

## Methods

### Study design

The study was designed as a non-randomised, controlled intervention trial aiming to improve NCD risk profile due to lifestyle changes with a total duration of 24 months (2018–2020). Data were assessed at six time points: baseline (*t*_0_), 10 weeks (*t*_1_), 6 months (*t*_2_), 12 months (*t*_3_), 18 months (*t*_4_) and 24 months (*t*_5_). Participants completed questionnaires to assess socio-demographics and health-related parameters such as health behaviour, health economic parameters (for the calculation of direct and indirect costs), quality of life, physical activity, and well-being. In addition, dietary intake was assessed using a semi-quantitative 3-day record. Blood parameters (e.g. cholesterol, triglycerides, or fasting glucose), anthropometric parameters (e.g. weight, height, or waist circumference) and vital signs (systolic and diastolic blood pressure) were collected during health check-ups at each measurement time point. Blood was sampled in fasted state and analysed at the University Hospital of Muenster. Due to the COVID-19 pandemic and contact restrictions, the health check after 24 months (*t*_5_) could not be conducted in person. The questionnaires were sent to the participants by postal service. Therefore, for this time point only questionnaire data are available. In the intervention group (IG) the HLCP-2 was implemented, the control group (CG) received no intervention. For ethical reasons, the participants of the CG were informed about their personal health check results. Blinding of participants or instructors was not possible by reason of the study design (as described previously: Koeder et al., 2021a). However, staffs performing laboratory tests were unaware of group allocations. Randomisation of participants was not possible due to the complex real-world approach. Real-world laboratories are a way to translate science into practice by conducting interventions not in a clinical or lab setting but in a real-world context (Renn, 2018) (as described previously: Koeder et al., 2021a). Due to limited time and staff, the CG started 6 months later than the IG (start: April [IG] and October [CG]). The follow-up durations were equal in both groups. The study was registered in the German Clinical Trial Register (DRKS; reference: DRKS00018775; www.drks.de).

### Participants

**Figure 1.**
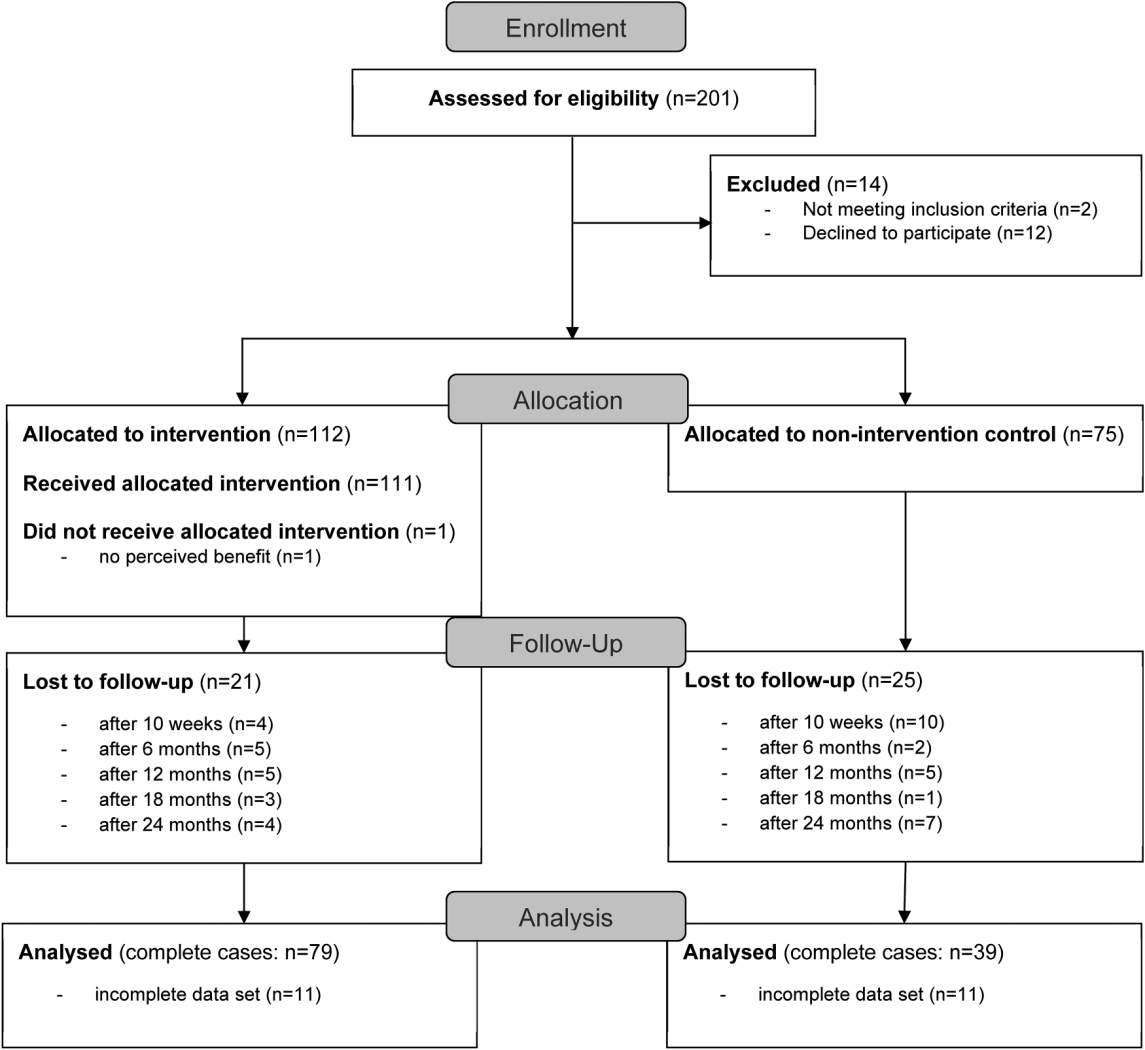
CONSORT participants’ flow diagram; participants categorised as “lost to follow-up” did not show up to health checks or withdrew from the study.

Due to the real-world approach, people were recruited from the general population. Recruitment of participants was performed in two different communities in Germany, an “intervention municipality” and a “control municipality”. Therefore, two comparable rural communities in the same region (north-west Germany) were selected to ensure that participants of the CG were unaware of the contents of the intervention. Inclusion criteria were adult age (≥18 years) as well as physical and mental ability to participate in the study. Figure 1 shows the allocation of the participants to the study groups. In total *n* = 201 participants were enrolled in the study, but *n* = 14 were excluded prior to baseline because they did not meet inclusion criteria or declined to participate. Subsequently *n* = 112 were allocated to the IG and *n* = 75 to the CG. Posters, newspaper articles and flyers were distributed in both communities and a health market was conducted in the IG in cooperation with local stakeholders. Before being included in the study, the participants gave their written informed consent. The study was conducted in accordance with the Declaration of Helsinki, and the protocol was approved by the ethics committee of the Medical Association of Westphalia-Lippe and of the University of Muenster (Muenster, Germany; reference: 2018-171-f-S; approved 4 April 2018).

### Intervention

The HLCP-2 included a 10-week intensive intervention phase. It consisted of 14 consecutive seminars twice a week, which lasted two hours each. Additionally, eight workshops with a smaller group size (20 participants) were conducted (e.g. cooking classes, shopping tours, sports, and relaxation courses). The four basic principles of the intervention were a healthy, plant-based diet (Jayedi et al., 2020), physical activity (Posadzki et al., 2020), stress management (O'Connor et al., 2021) and community support (Berra et al., 2017). The seminars covered, among other things, various symptoms, and the typical progression of NCDs such as diabetes mellitus type 2 and coronary heart diseases, but also topics such as the influence of dietary fat quality, cholesterol, and high blood pressure on the risk profile for NCDs and were based on the CHIP approach (Englert et al., 2007; Wennehorst et al., 2016). Dietary recommendations included a diet high in vegetables and fruits, whole grains, legumes, nuts, seeds, healthy oils, and low in meat, high-fat diary, highly processed foods, and salt (Jayedi et al., 2020). Each participant was offered two one-on-one coaching sessions (at baseline and after 10 weeks). Participants received a healthy lifestyle handbook, a recipe booklet, and a one-pager with an overview of the key lifestyle recommendations. Subsequently, a less intensive alumni phase of 22 months duration followed, where participants attended monthly seminars, and an e-mail newsletter was sent to refresh content of the intensive phase and support long-term behaviour changes. The participants of the IG had the opportunity to participate in all events of the intervention free of charge (health checks, seminars, workshops, and alumni meetings) and both groups were informed about the results of the health checks without any costs. In the CG, restaurant vouchers were additionally raffled at each time point as an incentive.

### Data collection

In the questionnaire about socioeconomic and health economic data, participants reported regular medication use. They were asked to specify drug names, dose, dosage throughout the day, as well as the form of intake (tablets, drops, etc.). Use and dose of the medication could only be prescribed or changed by their personal physician. We noticed that some participants were unaware of their medical condition, thus a diagnosis was added by hand when associated medication was taken. The drugs indicated in the questionnaire by the participants were assigned to drug families based on an index from Germany (Rote Liste®) (Rote Liste Service GmbH, 2021). The group of GLM included both oral antidiabetic agents and insulin. AHD comprised for example diuretics as well as beta-receptor and calcium channel blockers*.* Timolol hydrogen maleate is classified as a beta-blocker, but considering its usage for the treatment of glaucoma, it was disregarded in the analysis. Drugs from the groups of HMG-CoA reductase inhibitors and azetidones were included for the treatment of dyslipidemia. To determine the reduction or increase in medication use, we considered and categorised the number and dosage of medication over the course of the study. For accuracy, all analysed data were entered twice.

Medication costs were calculated using a German medication index named Lauer-Taxe® (CompuGroup Medical, n.d.). Manufacturer and pharmacy discounts were subtracted from the pharmacy retail price. Individual discounts of health insurance companies could not be considered, because they can vary regionally and are not publicly available. Additional payments from patients were not taken into account, as they are not relevant from a social perspective (Schwalm et al., 2020). Calculation of costs was based on the indicated medications per day and extrapolated to costs per week for better comparability. If a drug was available in different package sizes, the one with the lowest costs per drug was selected. In case of missing information, assumptions had to be made: If the brand was not specified by the participants, the most commercial product was chosen; missing information on dosage was estimated by using available data from the other measurement time points or using standard dosage.

### Study hypothesis

Body weight was the primary outcome parameter of the study. We hypothesised that participants receiving the HLCP-2 would reduce significantly more weight during the study period compared to baseline parameters and significantly more than the ones not receiving an intervention. The results of the primary outcome parameter showed a significant reduction of body weight after 10 weeks and 1 year compared to baseline within the IG and in the between-group comparison (Koeder et al., 2021b). Medication use and medication costs for diabetes, hypertension, and dyslipidemia were secondary outcome parameters of the study. Additionally, these medications were summarised as NCD medications and analysed separately. Simultaneously the secondary hypothesis states a significant reduction of medication use and costs for the IG at the measurement time points (*t*_1_–*t*_5_) compared to baseline and control.

### Statistical analysis

An adequate sample size was determined based on the primary outcome parameter (change in body weight; as described previously: Koeder et al., 2021b). The analysis was performed using a predefined analysis plan. All analyses were performed on a complete cases basis (CCA). In the descriptive statistics, categorial data were reported in absolute numbers and percentage (%). Quantitative variables were expressed in means ± standard derivation (SD). To test data for normality, Shapiro–Wilk test was used, with *p* < 0.05 describing a non-normal distribution.

Within-group changes were evaluated using the paired t-test for normally distributed and Wilcoxon-signed rank test for non-normally distributed variables. Independent t-tests compared the between-group difference of normally distributed continuous variables, while non-normally distributed values were evaluated using the Mann–Whitney *U* test. Fisher`s exact test was performed for between-group differences of categorial variables. All tests were two-sided.

Multiple linear regression (MLR) models were used for covariate-adjusted comparison the intervention and CG regarding medication use and costs. Confounders were identified and considered as potential covariates in the multiple regression models. Forward-backward selection was performed to find final models that were statistically significant (general linear *F*-test: *p* ≤ 0.05), have the highest corrected *R*^2^ and the fewest covariates. Residuals were inspected to check for normality. For all analyses, results were considered significant at *p* < 0.05. SPSS version 27 for Windows was used (SPSS Inc., Armonk, NY, USA).

## Results

A total of 118 participants (IG: *n* = 79; CG: *n* = 39) completed data sets for all measurement time points (baseline, 10 weeks as well as 6, 12, 18 and 24 months). A participants’ flow diagram shows the study process from enrollment to analysis in Figure 1.

### Baseline characteristics

Table 1 shows the baseline characteristics of both study groups. Study participants were, on average, overweight (BMI*:* IG:27.4 ± 0.6 kg/m^2^; CG: 28.1 ± 0.9 kg/m^2^), middle-aged (age at baseline*:* IG: 59.2 ± 0.9 years; CG: 56.4 ± 1.5 years), and female (IG: 70.9%; CG: 61.5%). Participants who were part of the IG were older (*p* = 0.031) and had a higher education level (*p* = 0.019) than members of the CG. There were no statistically significant differences between both groups in blood parameters and vital signs at baseline. Medication use of AHD, GLM and LLD was also comparable at baseline between groups.

**Table 1.** Baseline characteristics by study groups (complete cases).

Variable	Intervention group (*n* = 79)	Control group (*n* = 39)	*p*-Value
**Age, years: mean ± SD**	59.2 ± 7.8	56.4 ± 9.2	**0** **.** **031** ^c^
**Sex, *n* (%)**			0.402^a^
Male	23 (29.1)	15 (38.5)	
Female	56 (70.9)	24 (61.5)	
**Body weight, kg: mean ± SD**	80.7 ± 19.0	83.9 ± 18.1	0.260^c^
**BMI, kg/m^2^: mean ± SD**	27.4 ± 5.3	28.1 ± 5.7	0.542^c^
**Waist circumference, cm: mean ± SD**	97.5 ± 15.1	96.8 ± 14.8	0.975^c^
**Education level, *n* (%)**			**0**.**019**^a^
Lower secondary school	14 (17.7)	14 (35.9)	
Secondary school	36 (45.6)	12 (30.8)	
University entrance qualification	14 (17.7)	11 (28.2)	
University degree	15 (19)	2 (5.1)	
**Marital status, *n* (%)**			0.547^a^
Married	63 (79.7)	35 (89.7)	
Partner, unmarried	5 (6.3)	1 (2.6)	
Single (not widowed)	7 (8.9)	1 (2.6)	
Single (widowed)	4 (5.1)	2 (5.1)	
**Diagnose, *n* (%)**			
Hypertension	35 (44.3)	18 (46.2)	1.000^a^
Diabetes mellitus	3 (3.8)	2 (5.1)	1.000^a^
Dyslipidemia	18 (22.8)	6 (15.4)	0.467^a^
**Vital signs: mean ± SD**			
Systolic BP, mm Hg	133.3 ± 15.0	134.9 ± 14.5	0.585^b^
Diastolic BP, mm Hg	81.3 ± 9.0	80.7 ± 9.3	0.636^c^
**Blood parameters: mean ± SD**			
TC, mg/dL	209.2 ± 38.0	205.9 ± 44.8	0.677^b^
HDL-C, mg/dL	66.6 ± 18.9	62.1 ± 17.9	0.226^c^
LDL-C, mg/dL	134.1 ± 36.2	138.5 ± 41.5	0.555^b^
TG, mg/dL	104.6 ± 52.7	112.6 ± 49.3	0.292^c^
Fasting glucose, mg/dL	98.3 ± 11.5	102.0 ± 12.5	0.352^c^
HbA1c, %	5.4 ± 0.5	5.5 ± 0.4	0.691^c^
**Medication use, *n* (%)**			
Antihypertensive drugs	30 (38)	16 (41)	0.842^a^
Glucose-lowering medications	0 (0)	2 (5.1)	0.107^a^
Lipid-lowering drugs	14 (17.7)	6 (15.4)	1.000^a^
**Smoking status, *n* (%)**			0.182^a^
Current/occasional	8 (10.1)	9 (23.1)	
Ex	28 (35.4)	13 (33.3)	
Never	43 (54.4)	17 (43.6)	

SD: standard deviation; BMI: body mass index; BP: blood pressure; TC: total cholesterol; LDL-C: LDL cholesterol; HDL-C: HDL cholesterol; TG: triglycerides.

^a^ Fisher's exact test (two-sided).

^b^ Independent *t*-test (two-sided).

^c^ Mann–Whitney *U* test (two-sided). Significant values are marked in bold.

### Descriptive results

During the intensive phase of the study, seminar attendance was high. Overall, 95% of the analysed participants (*n* = 79) attended in at least half of the seminars and 63.3% participated in at least 12 of the 14 seminars.

Table 2 indicates medication use for the treatment of NCDs during the study period. Subsequently, the medications are shown divided according to GLM, AHD and LLD. At baseline, 44% of participants (*n* = 35) in the IG and 47% (*n* = 18) in the CG were taking medication for at least one of the NCDs described. Among the participants in the IG 56% (*n* = 44) were taking no medication for NCD, 39% (*n* = 31) one or two drugs per day and 5% (*n* = 4) three or more drugs per day. In comparison, in the CG 53% of the participants (*n* = 20) were taking no medication, 26% (*n* = 10) were taking one or two drugs and 21% (*n* = 8) three or more drugs for NCDs. During the study period, the proportion of participants taking NCD medication in the IG first decreased and then slightly increased again. In comparison, in the CG the number of participants taking NCD medication increased during the complete study period.

**Table 2.** Medication use during the study period (*t*_0_–*t*_5_) in the IG (*n* = 79) and CG (*n* = 39).

		*t*_0_	*t*_1_	*t*_2_	*t*_3_	*t*_4_	*t*_5_
Medication for NCDs	IG	35 (44.3%)	33 (41.8%)	32 (40.5%)	34 (43.0%)	33 (41.8%)	36 (45.6%)
	CG	18 (46.2%)	19 (48.7%)	23 (59.0%)	21 (53.8%)	22 (56.4%)	23 (59.0%)
Reduction	IG	N/A	7 (8.9%)	12 (15.2%)	10 (12.7%)	14 (17.7%)	14 (17.7%)
	CG	N/A	0 (0.0%)	0 (0.0%)	5 (12.8%)	4 (10.3%)	3 (7.7%)
Increase	IG	N/A	2 (2.5%)	6 (7.6%)	6 (7.6%)	7 (8.9%)	11 (13.9%)
	CG	N/A	2 (5.1%)	7 (17.9%)	8 (20.5%)	11 (28.2%)	11 (28.2%)
Glucose-lowering medications (GLM)	IG	0 (0.0%)	0 (0.0%)	0 (0.0%)	0 (0.0%)	0 (0.0%)	1 (1.3%)
	CG	2 (5.1%)	2 (5.1%)	2 (5.1%)	2 (5.1%)	2 (5.1%)	2 (5.1%)
Reduction	IG	N/A	0 (0.0%)	0 (0.0%)	0 (0.0%)	0 (0.0%)	0 (0.0%)
	CG	N/A	0 (0.0%)	0 (0.0%)	0 (0.0%)	0 (0.0%)	0 (0.0%)
Increase	IG	N/A	0 (0.0%)	0 (0.0%)	0 (0.0%)	0 (0.0%)	1 (1.3%)
	CG	N/A	0 (0.0%)	0 (0.0%)	0 (0.0%)	0 (0.0%)	0 (0.0%)
Antihypertensive drugs (AHD)	IG	30 (38.0%)	28 (35.4%)	27 (34.2%)	27 (34.2%)	27 (34.2%)	27 (34.2%)
	CG	16 (41.0%)	17 (43.6%)	20 (51.3%)	19 (48.7%)	20 (51.3%)	22 (56.4%)
Reduction	IG	N/A	7 (8.9%)	9 (11.4%)	9 (11.4%)	14 (17.7%)	13 (16.5%)
	CG	N/A	0 (0.0%)	0 (0.0%)	4 (10.3%)	3 (7.7%)	3 (7.7%)
Increase	IG	N/A	2 (2.5%)	5 (6.3%)	5 (6.3%)	7 (8.9%)	7 (8.9%)
	CG	N/A	2 (5.1%)	5 (12.8%)	4 (10.3%)	10 (25.6%)	9 (23.1%)
Lipid-lowering drugs (LLD)	IG	14 (17.7%)	14 (17.7%)	13 (16.5%)	14 (17.7%)	14 (17.7%)	14 (17.7%)
	CG	6 (15.4%)	6 (15.4%)	8 (20.5%)	7 (17.9%)	7 (17.9%)	8 (20.5%)
Reduction	IG	N/A	1 (1.3%)	3 (3.8%)	2 (2.5%)	1 (1.3%)	3 (3.8%)
	CG	N/A	0 (0.0%)	0 (0.0%)	2 (5.1%)	1 (2.6%)	0 (0.0%)
Increase	IG	N/A	0 (0.0%)	2 (2.5%)	2 (2.5%)	0 (0.0%)	3 (3.8%)
	CG	N/A	0 (0.0%)	2 (5.1%)	4 (10.3%)	3 (7.7%)	4 (10.3%)

IG: intervention group; CG: control group; N/A: not applicable.

Considering only the complete cases, there was no person taking GLM in the IG at baseline. At the last measurement time point one person in the IG started taking medication*.* In the CG two people were steadily taking GLM and none of them reduced their daily dosage.

In the IG 30 participants were taking AHD at baseline, *n* = 13 (17%) were told by their personal physician to reduce the dosage of their medication after two years compared to baseline, while about *n* = 7 (9%) had to increase their dosage. In contrast, in the CG of the 16 participants taking AHD at baseline, *n* = 9 (23%) had to increase the dosage or started taking medication after two years. During this period, only *n* = 3 (8%) of the CG were advised to reduce medication use.

During the study period LLM use remained the same in the IG, with one exception after 12 months. In the CG, *n* = 6 (15%) were taking medication for dyslipidemia at baseline, while *n* = 8 (21%) were taking them after 24 months. In addition, *n* = 4 (10%) of the subjects in the CG had to increase the dose of medication during the study period.

### Calculation of costs

Table 3 describes the costs per week (in €) expended for the drugs analysed for all measurement time points compared to baseline. On average, 4.16 ± 3.9€ was spent on drugs for the treatment of NCDs in the IG at baseline. The costs within the IG decreased at all measurement time points compared to baseline without reaching statistical significance. In comparison, 5.17 ± 5.1€ were spent in the CG at baseline. Costs within the CG increased significantly after 6 months (*p* = 0.009), 18 months (*p* = 0.012) and 24 months (*p* = 0.003) compared to baseline. Change of costs differed significantly in the IG compared to CG during the study period at all time points except for the second time point after 10 weeks (*t*_0_–*t*_2_: *p* = 0.004; *t*_0_–*t*_3_: *p* = 0.040; *t*_0_–*t*_4_: *p* = 0.003; *t*_0_–*t*_5_: *p* = 0.008).

**Table 3.** Medication costs (in €) per week during the study period (*t*_0_–*t*_5_) in the IG and CG.

			*t*_0_	*t*_1_	*p*-Value^a^	*t*_2_	*p*-Value^a^	*t*_3_	*p*-Value^a^	*t*_4_	*p*-Value^a^	*t*_5_	*p*-Value^a^
Medication for NCDs	IG (*n* = 41)	Mean ± SD	4.16 ± 3.9	3.92 ± 3.8	0.103	3.78 ± 3.7	**0** **.** **004**	3.90 ± 3.8	**0**.**040**	3.70 ± 3.7	**0**.**003**	3.75 ± 3.3	**0**.**008**
		Sum ± change	170.62	160.75 − 9.87		155.11 − 15.51		160.08 − 10.54		151.73 − 18.89		153.95 − 16.67	
	CG (*n* = 23)	Mean ± SD	5.17 ± 5.1	5.28 ± 4.5		6.18* **±** **4.7**		6.16 ± 4.9		6.55* **±** **4.8**		6.66** **±** **4.8**	
		Sum ± change	119.02	121.39 + 2.37		**142.19** **+ 23.17**		141.77 + 22.75		**150.72 + 31.70**		**153.17 + 34.15**	
Glucose-lowering medications	IG (*n* = 1)	Mean ± SD	0.00	0.00	0.480	0.00	0.480	0.00	1.000	0.00	0.480	2.24	0.157
		Sum ± change	0.00	0.00 ± 0.00		0.00 ± 0.00		0.00 ± 0.00		0.00 ± 0.00		2.24 + 2.24	
	CG (*n* = 2)	Mean ± SD	10.36 ± 8.1	7.31 ± 3.8		10.29 ± 8.0		10.36 ± 8.1		10.29 ± 8.0		10.36 ± 8.1	
		Sum ± change	20.72	14.62 − 6.10		20.58 − 0.14		20.72 ± 0.00		20.58 − 0.14		20.72 ± 0.00	
Antihypertensive drugs	IG (*n* = 32)	Mean ± SD	4.29 ± 3.6	3.98 ± 3.5	0.067	3.91 ± 3.5	**0**.**006**	3.89 ± 3.5	0.052	3.73 ± 3.3	**0**.**004**	3.64 ± 3.2	**0**.**006**
		Sum ± change	137.19	127.32 − 9.87		125.18 − 12.01		124.33 − 12.86		119.41 − 17.78		116.60 − 20.59	
	CG (*n* = 22)	Mean ± SD	3.81 ± 3.5	4.19 ± 3.9		4.64* **±** **3.4**		4.73 ± 3.5		5.12* **±** **3.6**		5.18** **±** **3.3**	
		Sum ± change	83.81	92.28 + 8.47		**102.01 + 18.20**		104.04 + 20.23		**112.64 + 28.83**		**113.87 + 30.06**	
Lipid-lowering drugs	IG (*n* = 18)	Mean ± SD	1.86 ± 1.1	1.86 ± 1.1	1.000	1.66 ± 1.3	0.187	1.99 ± 1.3	0.860	1.80 ± 1.3	0.106	1.95 ± 1.1	0.254
		Sum ± change	33.43	33.43 ± 0.0		29.93 − 3.50		35.75 + 2.32		32.32 − 1.11		35.11 + 1.68	
	CG (*n* = 9)	Mean ± SD	1.61 ± 1.4	1.61 ± 1.4		2.18 ± 1.1		1.90 ± 1.3		1.94 ± 1.3		2.07 ± 1.1	
		Sum ± change	14.49	14.49 ± 0.0		19.60 + 5.11		17.01 + 2.52		17.50 + 3.01		18.59 + 4.10	

Wilcoxon-test for within-group differences with *p < 0.05. **p < 0.01 for within-group comparison to baseline; Mann–Whitney *U* test for between-group differences.

^a^ *p*-Value for between-group comparison of the change of costs (reference: *t*_0_). Significant values are marked in bold.

NCD: non-communicable diseases; IG: intervention group; CG: control group; SD: standard derivation.

**Figure 2.**
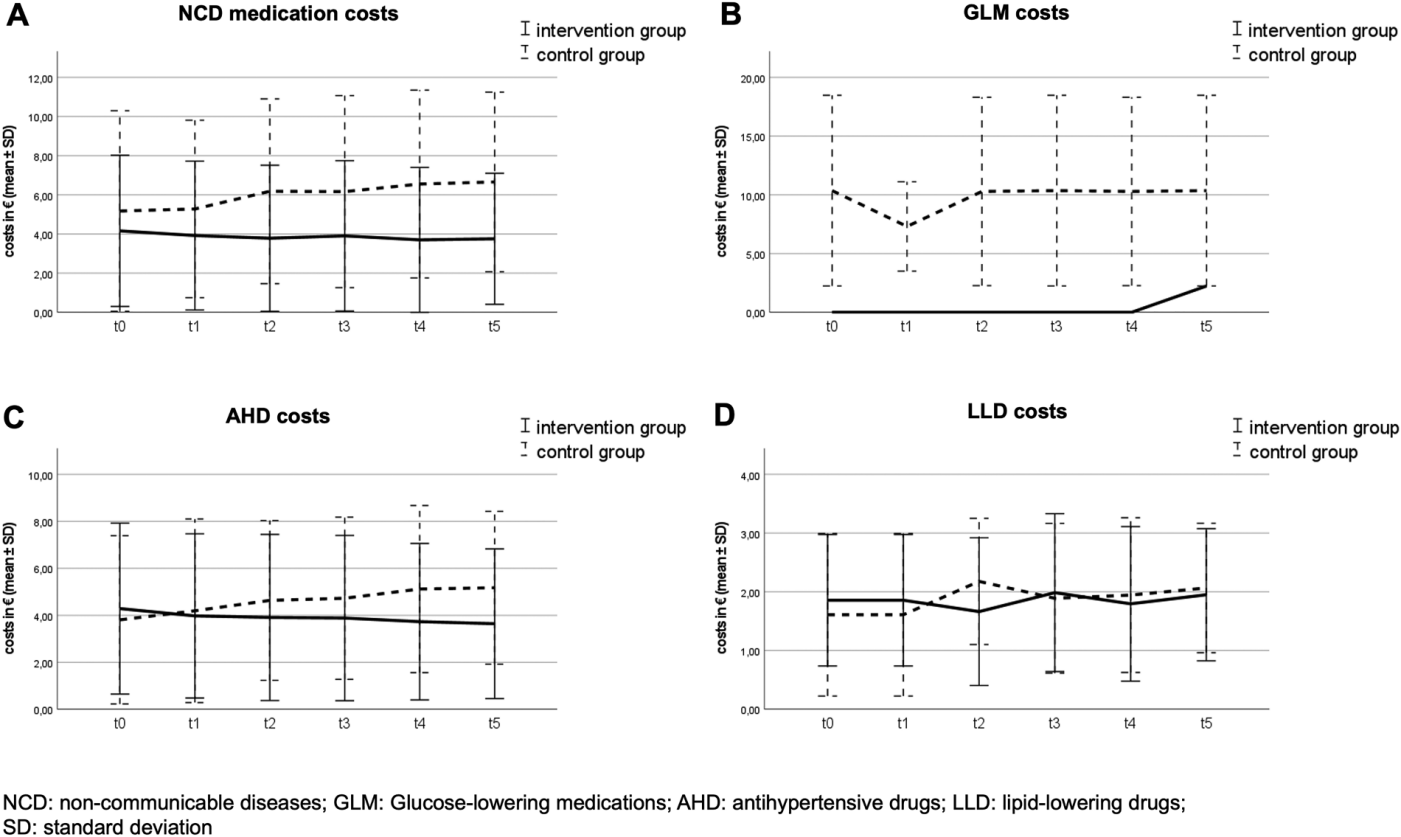
Change of medication costs during the study period (*t*_0_–*t*_5_).

Change of costs for AHD differed significantly between IG and CG at 6 months (*p* = 0.006), 18 months (*p* = 0.004) and 24 months (*p* = 0.006). Due to the small number of participants diagnosed with diabetes mellitus, no significant cost changes for GLM were observed. LLD costs did not change significantly neither. Within the IG, a decrease in costs was observed after 6 months, although it did not reach statistical significance (*p* = 0.143). Figure 2 illustrates the change of costs during the study period divided by medication groups.

### Multiple linear regression analysis

To adjust for group differences that appear due to a lack of randomisation, a MLR analysis was conducted (Table 4). The dependent variable, change of costs for NCD medication, differed significantly between the IG and the CG in all final models, that is at 10 weeks (*p* = 0.010), 6 months (*p* < 0.001), twelve months (*p* = 0.008), 18 months (*p* = 0.001) and 24 months (*p* = 0.001), which is consistent with the results of the descriptive statistic. In addition, having higher costs at baseline was a predictor for greater reduction in costs for NCD medication at all measurement time points (10 weeks: *p* = 0.007; 6 months: *p* < 0.001; 12 months: *p* = 0.028; 18 months: *p* = 0.003; 24 months: *p* < 0.001). The MLR showed that being female had a significant influence on reduction of medication costs at 24 months (*p* = 0.022). In the final regression models vital parameters, blood lipid levels, anthropometric parameters, education level, marital status or smoking status were not shown to be independent predictors with a significant influence on medication costs for NCDs.

**Table 4.** Multiple regression analysis of the change in medication costs for NCDs after 10 weeks, 6, 12, 18 and 24 months (CCA, *n* = 64).

	*β*	SE	*p*-Value
**10 weeks** ^a^			
Constant (*β*_0_)	1.708	2.143	0.429
Group (ref. intervention)	0.807	0.304	**0** **.** **010**
Medication costs NCD at baseline	−0.114	0.041	**0**.**007**
HbA1c, %	−1.004	0.303	**0**.**002**
Age	0.065	0.018	**0**.**001**
**6 months** ^b^			
Constant (*β*_0_)	−3.282	1.629	**0**.**048**
Group (ref. intervention)	1.766	0.462	**<0**.**001**
Medication costs NCD at baseline	−0.192	0.054	**<0**.**001**
Age	0.060	0.027	**0**.**031**
**12 months** ^c^			
Constant (*β*_0_)	0.261	0.375	0.489
Group (ref. intervention)	1.372	0.498	**0**.**008**
Medication costs NCD at baseline	−0.125	0.555	**0**.**028**
**18 months** ^d^			
Constant (*β*_0_)	0.415	0.455	0.364
Group (ref. intervention)	2.052	0.602	**0**.**001**
Medication costs NCD at baseline	−0.211	0.067	**0**.**003**
**24 months** ^e^			
Constant (*β*_0_)	−3.930	2.181	0.077
Group (ref. intervention)	1.749	0.524	**0**.**001**
Medication costs NCD at baseline	−0.351	0.062	**<0**.**001**
Fasting glucose	0.057	0.022	**0**.**011**
Gender (ref. male)	−1.198	0.510	**0**.**022**

Dependent variable: change in medication costs for NCDs (compared to baseline).

^a^ Corrected *R*^2^ = 0.394. FS, general linear *F*-Test: *p* < 0.001.

^b^ Corrected *R*^2^ = 0.241. FS, general linear *F*-Test: *p* < 0.001.

^c^ Corrected *R*^2^ = 0.130. FS, general linear *F*-Test: *p* = 0.005.

^d^ Corrected *R*^2^ = 0.215. FS, general linear *F*-Test: *p* < 0.001.

^e^ Corrected *R*^2^ = 0.416. FS, general linear *F*-Test: *p* < 0.001.

Significant values are marked in bold. All residuals are normally distributed; SE: standard error; ref.: reference group, FS: forward selection.

## Discussion

The HLCP-2 had a positive effect on medication use for NCDs and associated costs. The descriptive statistics showed that medication use improved moderately after 10 weeks, 6, 12 and 18 months, while medication use in the CG increased at all measurement time points. In the IG, reductions in medication costs could not reach a significant level at any measurement time point compared to baseline (within-group), whereas the costs for NCD medication within the CG increased significantly after 6, 18 and 24 months compared to baseline. We stated that the manifestation of NCDs such as hypertension, diabetes mellitus or dyslipidemia is more prevalent with increasing age and blood parameters are deteriorating on average. Analyses prove that prevalence of hypertension in Germany is twice as high in the age group over 65 years (men: 65.1%, women: 63.8%) than in the age group 45–64 years (men: 38.3%, women: 31.6%) (Neuhauser et al., 2017). In addition, studies show that blood pressure, cholesterol and fasting glucose levels increase on average with age (Singh et al., 2012). Systolic blood pressure is additionally affected by increasing arterial stiffness over lifetime (Steppan et al., 2011). A between-group comparison was conducted to test whether the lifestyle intervention could influence this general deterioration of health status. The change of costs differed significantly compared to CG after 6, 12, 18 and 24 months. The MLR showed a significant difference between the IG and CG in change of medication costs at all measurement time points. Consequently, lifestyle interventions can be considered successful if worsening NCD risk factors and therapeutic regimes can be prevented.

The observed changes were primarily due to AHD whereas medication use for diabetes or dyslipidemia remained almost unchanged (within- and between-group changes). Due to the small number of participants with diabetes mellitus, no conclusion can be drawn about the impact of the HLCP-2 on medication use and costs in diabetes. Among all participants only five people were diagnosed with diabetes mellitus and just three of them took GLM during the study period.

Multiple clinical trials have already shown that lifestyle interventions have a great potential to halt the general deterioration of health status with increasing age (Burke et al., 2005; Hamdy et al., 2008, 2017; Hamdy and Carver, 2008; Johansen et al., 2017; Kempf et al., 2017; Ziv et al., 2013). The Look AHEAD (Action for Health in Diabetes) study, a multi-centre, randomised controlled clinical trial, conducted a lifestyle intervention to achieve weight loss in patients with diabetes mellitus type 2 for prevention of future cardiovascular diseases (The Look AHEAD Research Group, 2007). The study reported significant reductions of NCD medication and consequently medication costs after 10 years (GLM: 17%, LLD: 6%, AHD: 6%) (Espeland et al., 2014). The HLCP-2 reduced costs of NCD medication (GLM, AHD and LLD) by about 10% after two years. The greatest reduction is attributable to AHM (about 15%), which is considerably higher than in the Look-AHEAD study. To analyse the impact of lifestyle interventions in real-world settings, community-based studies are more appropriate. They include local stakeholders and policy makers and focus their intervention on the general population (Renn, 2018). Englert et al. (2012) underlined this as in the Rockford CHIP (Coronary Health Improvement Project) participants were able to reduce their daily medication dosage of oral antidiabetic agents as well as insulin.

Lifestyle intervention programs hold great potential to achieve positive changes in terms of lifestyle, individual health, and quality of life. Thus the HLCP-2 was shown to be effective in reducing body weight, BMI, and waist circumference at 10 weeks and 12 months (Koeder et al., 2021b). Based on an improved risk profile for NCDs, medications can be discontinued. Drug withdrawal or dose reduction is not only beneficial from a health economic perspective, but also from a medical one (Burke et al., 2005). It is important to note that a reduction in medications does not automatically mean a reduction in costs, as some medications have the same price regardless of dosage or have minimal price differences (CompuGroup Medical, n.d.). Due to reduction of medication use, side effects that occur due to taking medication can be reduced or eliminated and quality of life can be improved (Burke et al., 2005). In addition, lifestyle modification does not only treat a specific disease, but all risk factors can be improved simultaneously and knowledge about the diseases may be increased (Ndejjo et al., 2021). Overall, lifestyle interventions are relevant to improve population health, reduce the burden for the health care system, and save costs. A complete health economic evaluation was conducted and will be published separately.

### Strengths and limitations

A strength of the HLCP-2 is the non-intervention CG and the complex real-world setting. Lifestyle interventions seem to have a low attendance and adherence, which influences the treatment effect (Burgess et al., 2017). However, in our study, attendance at the intensive phase seminars was high, which may have led to a better understanding and implementation of a healthy lifestyle among participants. The main limitation is the lack of randomisation (as described previously: Koeder et al., 2021a). Since the CG and the IG were located in two separated small municipalities in Germany, randomisation was not possible, which may have led to selection bias. However, the baseline characteristics of the IG and CG were similar except for age and educational level. In the MLR, we adjusted for age and education level, whereby neither parameter had a significant influence on the cost changes. The recruitment of the participants via health market could also contribute to selection bias, since primarily people who are already interested in health participated. A further limitation is that the onset of the CG was delayed. The CG started 6 months after the IG, but the course of the study was identical (same follow-up duration). Another limitation is the COVID-19 pandemic since planned health checks could not be realised, and the lifestyle of the participants was affected by lockdowns and contact restrictions (Bakaloudi et al., 2021). The limitations of the present study include the use of self-report questionnaires on health economic parameters, which might lead to response bias. Differences in sample size resulting from the sample size calculation of the primary parameter (change in body weight) could also be a limiting factor. The relatively small sample size can be explained by the complete-case analysis. The costs were calculated with data from Germany, but the cost factors of drugs and the structure of the health care system differ between countries. The results need to be tested for generalisability for other countries and health care systems (Palmer et al., 2004; Schwalm et al., 2020).

## Conclusion

The HLCP-2 resulted in a moderate reduction and prevented an increase in the use and costs of NCD-medication due to a healthy lifestyle. Costs were reduced compared to the CG, with the greatest impact on AHD. In the future, researchers should perform health economic evaluations of lifestyle intervention studies to further alert the health care system and policy makers to the benefits of lifestyle interventions.
